# Effect of two insect meals on the gut commensal microbiome of healthy sea trout (*Salmo trutta* vr. *trutta*)

**DOI:** 10.1186/s12917-023-03671-8

**Published:** 2023-08-14

**Authors:** Agata Józefiak, Mateusz Rawski, Bartosz Kierończyk, Damian Józefiak, Jan Mazurkiewicz

**Affiliations:** 1 Department of Preclinical Sciences and Infectious Diseases, Poznan University of Life Sciences, Wołyńska 35, 60-637 Poznań, Poland; 2 Division of Inland Fisheries and Aquaculture, Institute of Zoology, Poznan University of Life Sciences, Wojska Polskiego 71C, 60-625 Poznań, Poland; 3 Department of Animal Nutrition, Poznan University of Life Sciences, Wołyńska 33, 60-637 Poznań, Poland

**Keywords:** Sea trout, Next generation sequencing, NGS, Microbiome, Metagenome, Fish, Mealworm, Superworm

## Abstract

**Background:**

The balance of the intestinal commensal microbiome of fish and other animals plays an important role in the physiological processes of healthy animals, contributes to the defense against pathogens, stimulates the immune system and facilitates nutrient metabolism. In the last decade, the interest in the application of the insects in fish nutrition increased, although little is known regarding the effects of insect meals on the gastrointenstinal tract microbiome of the sea trout fingerlings. The aim of this study was to evaluate the effect of two diets containing mealworm (MW) and superworm (SW) on the microbiome of the digesta of sea trout fingerlings and the relative abundances of different taxa among communities under controlled conditions.

**Results:**

The insect meals produced a similar weight gain and survival rate to sea trout fed fishmeal. The most abundant bacterial phylum in all the treatment groups was Firmicutes followed by *Proteobacteria* and *Actinobacteria*, and significant differences in the amount of *Cyanobacteria* were observed in the SW group.

**Conclusions:**

The insect meals did not produce differences in the three most abundant phyla in the sea trout digesta. However, the effect of each type of meal on the lower taxonomic levels was evident, particularly in the case of the superworm meal. These microbiome differences indicated that mealworm meal was more related to fishmeal than superworm meal. Our results highlight the potential effects of insect meals, such as mealworm and superworm meals, on the microbiota of sea trout.

## Background

Fish, as well as other animals, must maintain the microbiome (bacteria, archaea, fungi, and viruses) in their intestinal tract in a balanced state, preserving the mutualistic relationship along their life cycles. The microbiome contributes to the defense against pathogens, stimulates the immune system and assists with nutrient metabolism [1–3]. In the last decade, the interest of the application of insects in fish nutrition has increased, although little is known about the effects of insect meals on the microbiome of the digesta of sea trout fingerlings.

The impact of insect products on gut health and microbiota has been analyzed with Rainbow trout (*O. mykiss*), Atlantic salmon (*Salmo salar*), Sea trout (*Salmo trutta* m*. trutta*), European sea bass (*Dicentrarchus labrax*), Gilthead seabream (*Sparus aurata*), Siberian sturgeon (*Acipenser baerii*), [4–14]. In the microbiota studies fish were fed mainly on *Hermetia illucens* (HI), *Tenebrio molitor* (TM), and *Musca domestica* (MD), and some studies investigated the effect of *Gryllus sigillatus* (GS)*, Blatta lateralis* (BL) and *Zophobas morio *(ZM) [15].

In general, insect meal does not adversely affect the gut morphology of fish. However, have been found that the inclusion of insects in fish diet can induce some mild gut histological changes. The mucosal and muscular layer thickening of the GIT in sturgeon fed both TM and HI [4]. Moreover, the dietary inclusion of insects increased submuscosa cellularity and production of neutral mucin in the proximal intestine of rainbow trout [8, 16].

Several studies have analyzed the fish microbiota using 16S rRNA gene amplicon sequencing. The microbial communities are affected by certain factors such as the species, the stage of development, the type of food consumed, and the intestinal morphology [4, 5]; environmental and physiological factors also modify the gut microbiota of fish [6]. The type of microbiome will also be conditioned by the feeding habit of the species; in salmonids such as rainbow trout, the predominant phyla are *Proteobacteria*, *Firmicutes*, *Bacteroidetes*, *Fusobacteria* and *Actinobacteria* [7, 8]. However, Rimoldi et al. (2018) found that rainbow trout fed higher levels of plant meals and rendered animal meals, and lower levels of fishmeal had higher quantities of *Fusobacteria* and *Bacteroidetes* in the intestine, and this difference was related to the lower growth performance [9]. Furthermore, Huyben et al. (2019) observed a variation in the abundance of a group of bacteria present in the gut of the rainbow trout according to the stage of development of the insect meal (larvae, prepupae, and pupae) [10]. The *Proteobacteria* and *Firmicutes* have been detected as dominated phyla in all gut regions of brown trout (*Salmo trutta* L.) [11]. Moreover, Michl et al. (2019) observed significantly increased abundances of *Proteobacteria* and *Fusobacteria* following the consumption of fishmeal, whereas plant-derived proteins increased the abundance of *Firmicutes* and *Bacterioidetes* [7].

In general, plant-based protein meals markedly modify the microbiome, as Kononova et al. (2019) showed the effects of soybean protein and carbohydrates, which are associated with some anti-nutritional factors, on the autochthonous microbiota, provoking inflammatory processes in the intestine of salmonids [12]. Regarding insect meals, Antonopoulou et al. (2019) found that rainbow trout fed 0 and 60% mealworm meal did not differ in the bacterial species or their amounts of relative abundance [13]. A possible explanation for this finding is that insects are part of the natural diet of this species.

Because insects are also part of the natural diet of sea trout, at least in the first stages of development, the aim of this study was to evaluate the effects of two insect meal diets on the microbiome of the digesta of sea trout fingerlings and the abundances of different taxa among communities.

## Results

### Growth performance

**Fig. 1:**
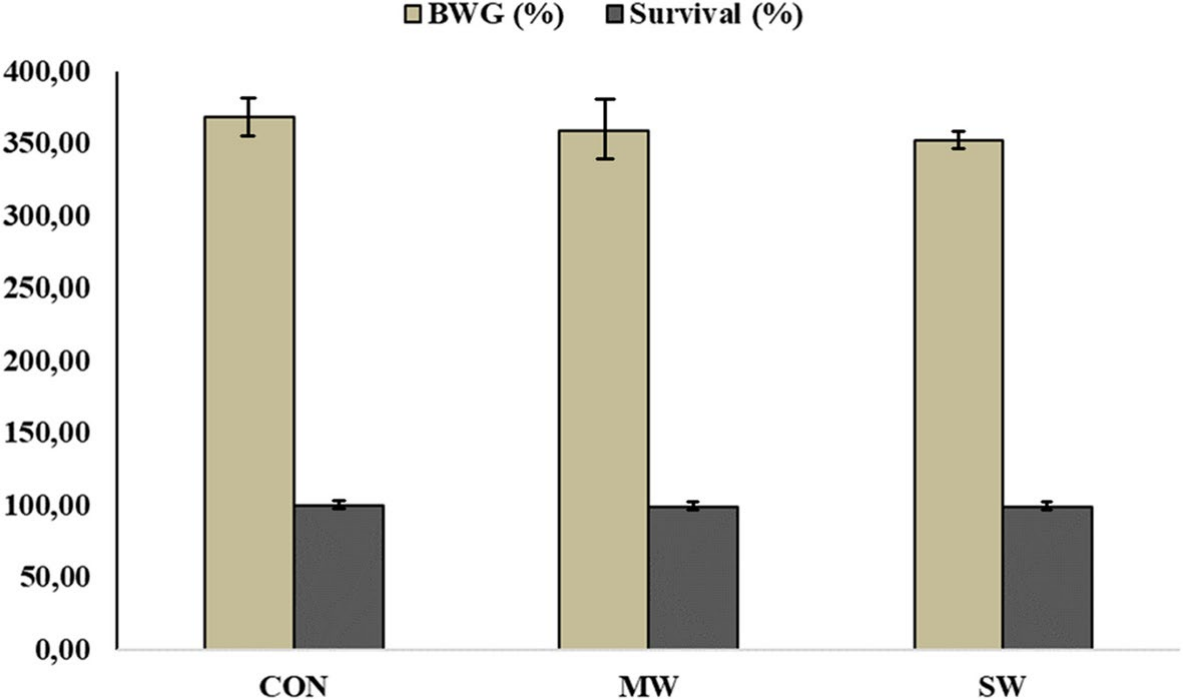
Bodyweight gain (BWG) and survival rate of sea trout at the end of the experiment. Experimental diets: fishmeal diet (CON), mealworm diet (MW), and superworm diet (SW)

At the end of the experimental period, no significant differences in body weight gain and survival rates were observed among groups fed the different experimental diets, as shown in Fig. 1.

### Microbiota diversity

**Fig. 2:**
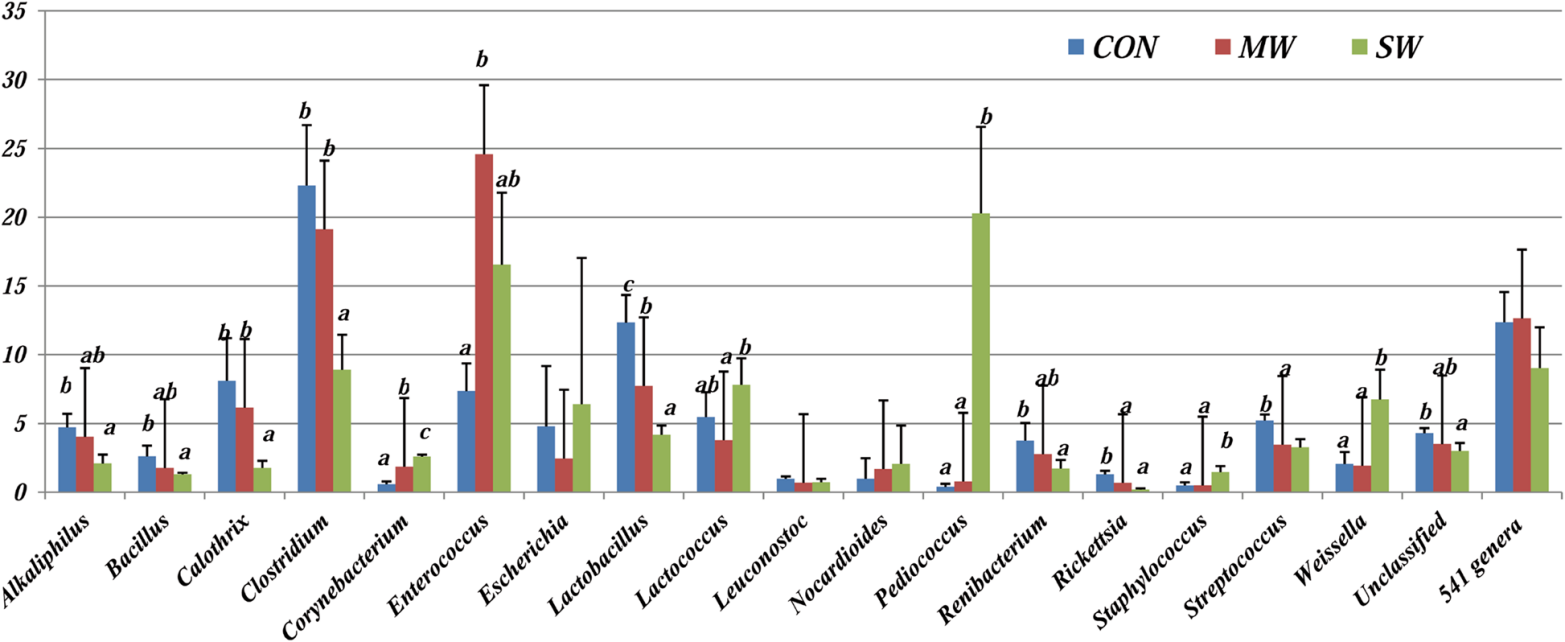
The relation between the genera proportions of sea trouts’ gut digesta in the different treatments: control (CON), mealworm meal (MW), and superworm meal (SW)

In general, 99.95% of the gut microbiota was constituted by bacteria, 0.03% by archaea and 0.01% by viruses, and 0.02% of the microorganisms were not identified. Considering bacteria, 24 phyla were identified, and 17 were represented with an abundance less than 1%, whereas the most predominant bacteria among the remaining phyla were *Firmicutes* (Fig. 2). In general, significant differences in the abundances of *Actinobacteria*, *Proteobacteria*, and Firmicutes were not observed, although the abundance of *Cyanobacteria* exhibited significant differences, as the SW group presented the lowest content of this phylum as well as the combined data for the 20 phyla (*p* < 0.05).

**Fig. 3:**
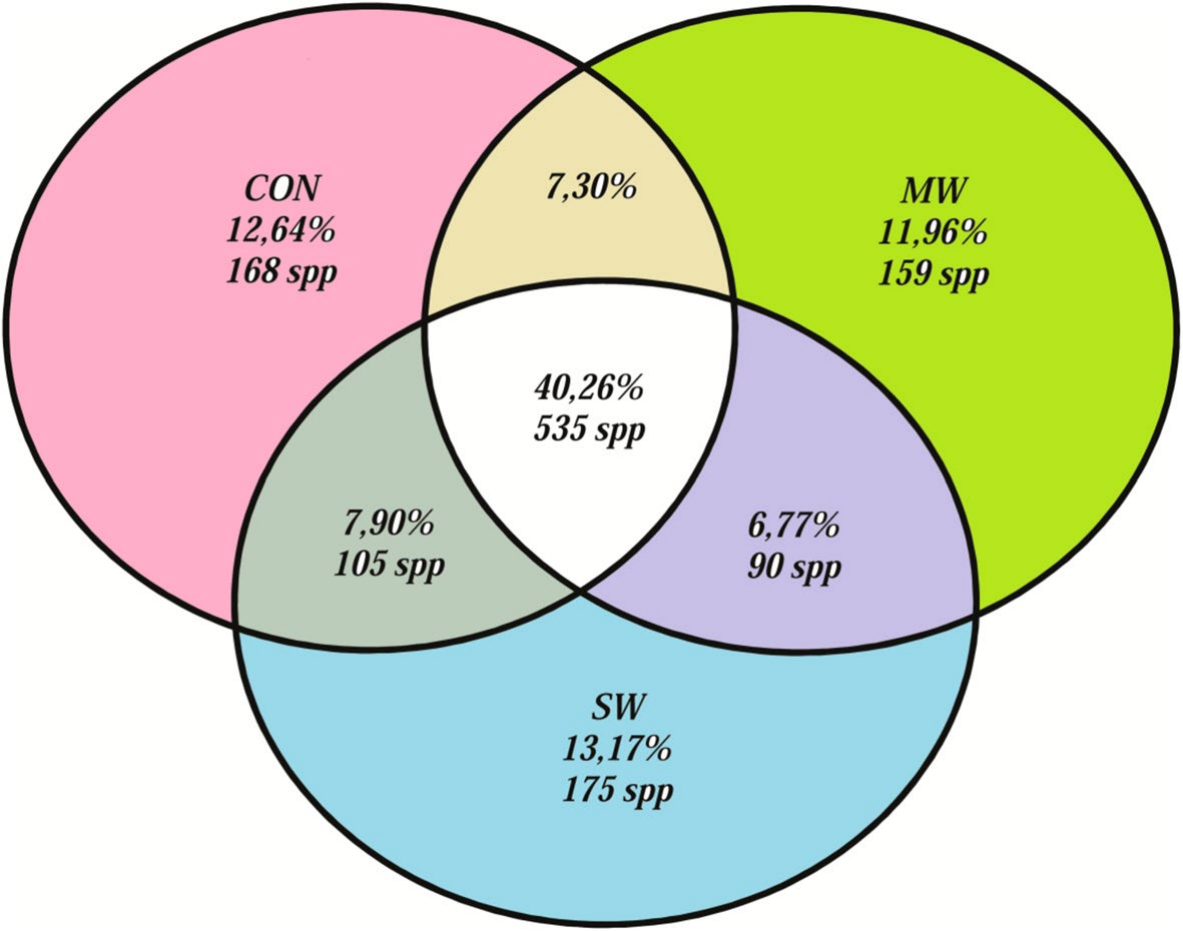
Relation of the number of species per treatment as well as those shared among treatments: Control diet (CON), mealworm diet (MW), and superworm diet (SW). The values are expressed in percentage and amount of species identified

Regarding the species relations (Fig. 3), 40.26% of the species were shared among treatments and 11.96 to 13.17% of the species are unique to each treatment; a lower number of shared species were observed between two treatments. Additionally, the amount of lactic acid bacteria (LAB) belonging to *Enterococcus, Pediococcus, Lactococcus*, and *Weisella* increased significantly in the fish fed the insect meals, namely, 36.16% and 47.98% in the MW and SW groups, respectively, compared to fish fed the control diet at 27.29% (*p* < 0.05).

Regarding species richness, 1328 species were identified, and the species richness was 905 species for the CON group, 879 species for the MW group, and 905 species for the SW group. At the same time, the Margalef index (D_Mg_) showed no significant differences among treatments. The alpha diversity indexes, such as Simpson Diversity Index (D), Menhinick index, and Shannon index (H), revealed that insect meals did not affect the species richness. Meanwhile, the Evenness index (e^H/S), and equitability Brillouin index also showed no significant differences among treatments. The dominance index (Berger-Parker) and Dominance D displayed similar values among meal-fed groups, and the dominance was low among groups. Regarding the abundance estimator, the Chao1 calculation showed no significant differences among treatments as well (Table 1).

**Table 1:** Alpha diversity indexes in sea trout gut microbiota species, comparison among treatments

	**CON**	**MW**	**SW**	**SEM**	***P*-value**
Individuals	73.50	74.00	74.50	0.78	0.6760
Dominance (D)	0.15	0.12	0.12	0.01	0.3782
Simpson 1-D +	0.85	0.88	0.88	0.01	0.3781
Shannon (H)	3.05	3.15	3.16	0.06	0.3632
Evenness e^H/S	0.04	0.05	0.05	2.1^–3^	0.1354
Brillouin	1.17	1.30	1.36	0.05	0.0966
Menhinick	51.05	49.28	50.80	1.69	0.7314
Margalef	118.58	114.28	117.63	4.09	0.7441
Equitability (J)	0.49	0.51	0.51	0.01	0.2154
Berger Parker	0.32	0.27	0.30	0.03	0.4398
Chao-1	510.50	492.75	508.00	16.88	0.7314

Values in the same row having different superscript letters are significantly different at *p* < 0.05, (*n* = 4)

**Fig. 4:**
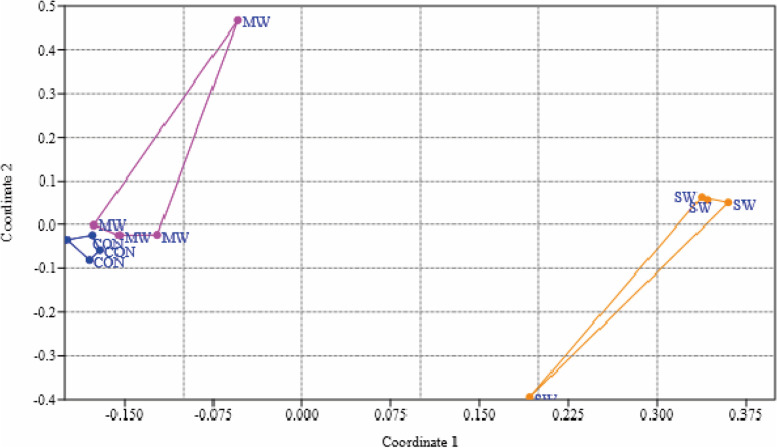
Non-metric multidimensional scaling (NMDS) analysis plot of gut microbiota of sea trouts fed with two experimental diets: CON (control diet), MW (mealworm diet), and SW (superworm diet)

**Fig. 5:**
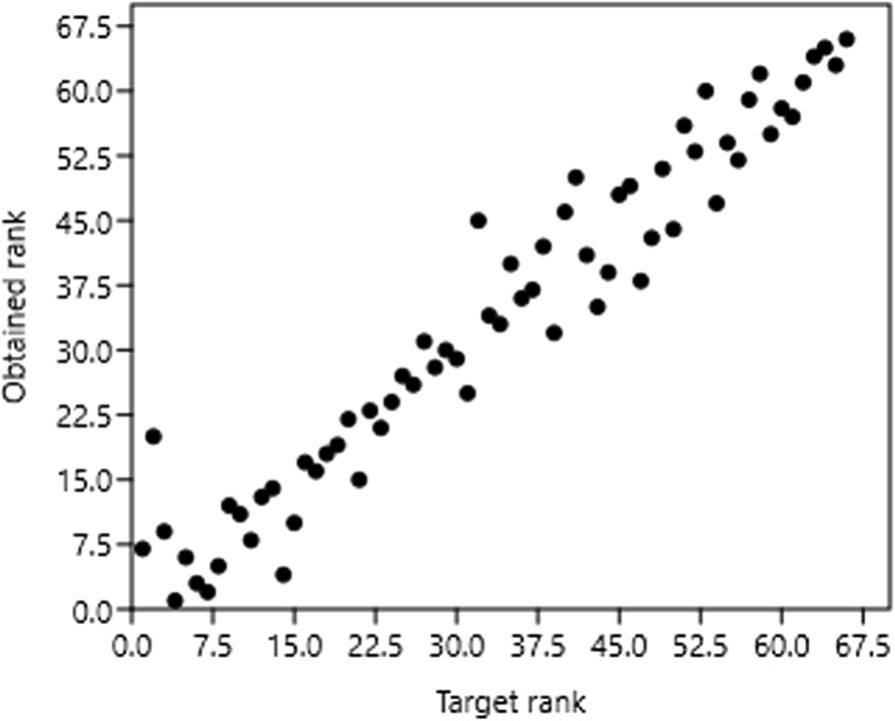
Shepard analysis plot and stress value of sea trouts’ microbiota fed with three experimental diets: CON (control diet), MW (mealworm diet), and SW (superworm diet)

**Fig. 6:**
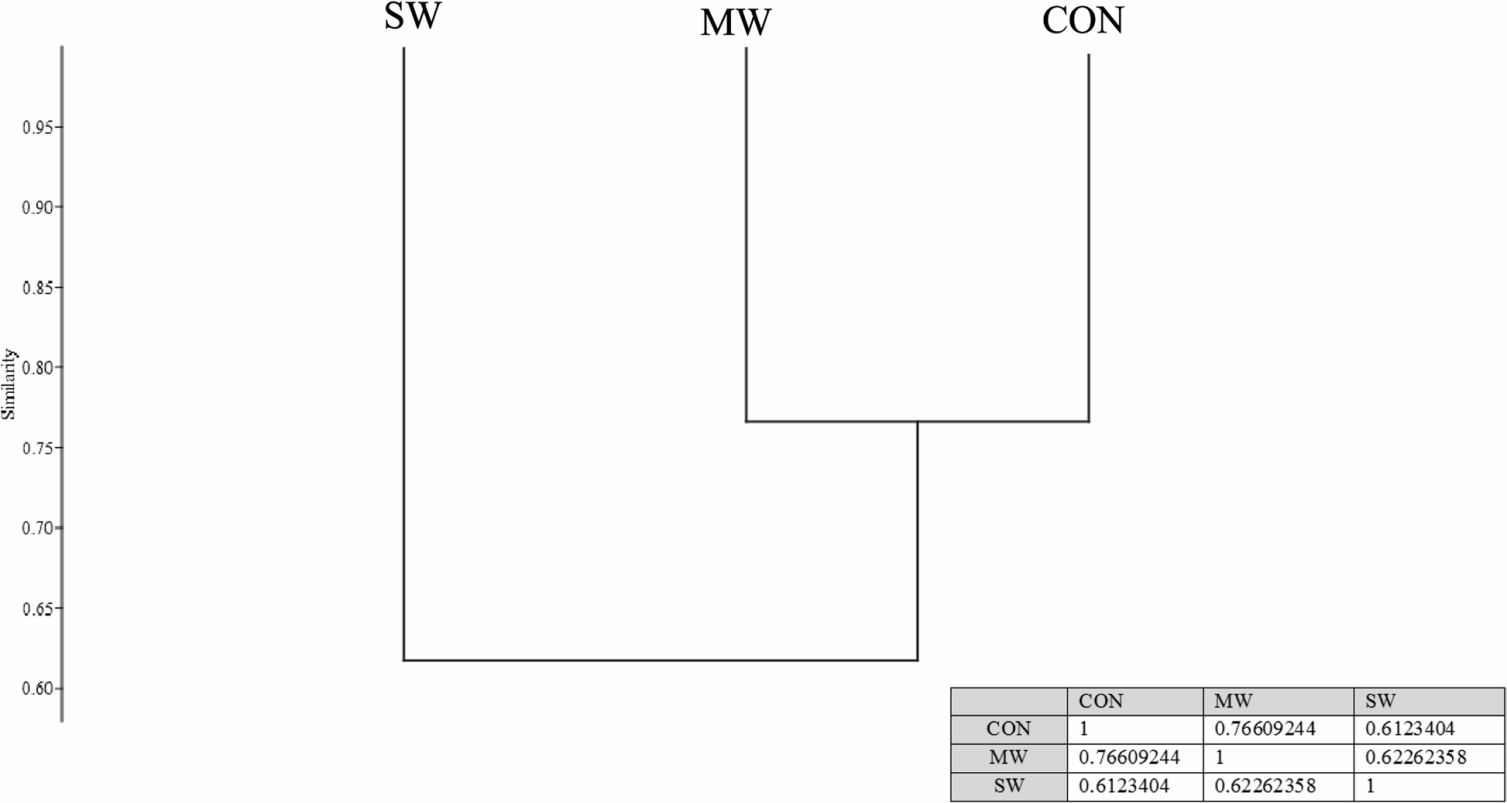
Sea trouts’ microbiota clusters similarities and dissimilarities between treatments; CON (control diet), MW (mealworm diet), and SW (superworm diet)

The Bray–Curtis analysis of beta diversity is presented in Table 2. Additionally, the nonmetric multidimensional scaling (NMDS) analysis (Fig. 4, and Fig. 5) and the clusters showed that CON and MW groups were much more related, with 76.6% similarity, than the SW group (61.2%) (Fig. 6).

**Table 2:** ANOSIM and Bray–Curtis results

ANOSIM	Bray–Curtis
Permutation N:	9999
Mean rank within:	14.67
Mean rank between:	40.56
R2:	0.787
p (same):	0.003

After observing the class distribution in sea trout digesta, *Bacilli* and *Clostridia* were the most predominant classes in the groups treated with the three experimental diets, although the SW group exhibited the highest percentage of *Bacilli*, but the lowest percentages of *Clostridia*, *Nostocophycideae* and other classes compared to groups fed the CON and MW diets (Table 3). A similar trend was observed for the class distribution, where *Bacilli* and *Clostridia* classes were the most predominant and the SW group exhibited the highest percentage of *Bacilli* and the lowest percentage of *Clostridia* among all three treatment groups. *Zophobas morio* meal also reduced the amount of *Nostocophycideae* and the grouped orders (Table 3). In terms of the family distribution, fish fed the SW diet exhibited lower percentages of *Bacillaceae*, *Clostridiaceae*, *Lachnospiraceae*, *Rickettsiaceae*, and *Rivulariaceae* than fish fed the CON and MW diets. In contrast, higher abundances of *Corynebacteriaceae*, *Lactobacillaceae*, and *Leuconostocaceae* were observed in the SW group than in fish fed the CON and MW diets. However, the percentage of *Enterococcaceae* was significantly lower in fish fed the CON diet those fed the SW and MW diets (*p* < 0.05).

**Table 3:** Relative abundance of the most dominant classes, orders, and families present in the digesta samples of sea trouts fed with the three experimental diets: control diet (CON), mealworm diet (MW), and superworm diet (SW)

Identifications	CON	MW	SW	SEM	*P*-value
**Classes**					
*Actinobacteria*	7.99	9.12	10.32	1.71	0.6415
*Alphaproteobacteria*	2.05	3.37	0.41	0.81	0.0826
*Bacilli*	41.57^a^	50.27^ab^	65.69^b^	5.47	0.0350
*Clostridia*	32.92^b^	26.77^b^	13.53^a^	2.75	0.0022
*Gammaproteobacteria*	5.43	2.97	7.43	3.74	0.7054
*Nostocophycideae*	8.24^b^	6.24^b^	1.80^a^	1.07	0.0060
40 classes^*^	1.79^c^	1.26^b^	0.78^a^	0.10	0.0003
**Order**					
*Actinobacteria*	7.99	9.12	10.32	1.71	0.6415
*Alphaproteobacteria*	2.05	3.37	0.41	0.81	0.0826
*Bacilli*	41.57^a^	50.27^ab^	65.69^b^	5.47	0.0350
*Clostridia*	32.92^b^	26.77^b^	13.53^a^	2.75	0.0022
*Gammaproteobacteria*	5.43	2.97	7.43	3.74	0.7054
*Nostocophycideae*	8.24^b^	6.24^b^	1.80^a^	1.07	0.0060
87 orders^*^	1.79^c^	1.26^b^	0.78^a^	0.10	0.0003
**Families**					
*Bacillaceae*	3.05^b^	2.00^a^	1.74^a^	0.25	0.0106
*Clostridiaceae*	27.70^b^	23.39^b^	11.22^a^	2.74	0.0056
*Corynebacteriaceae*	0.58^a^	1.85^b^	2.58^c^	0.13	0.0001
*Enterobacteriaceae*	5.26	2.77	7.31	3.73	0.6993
*Enterococcaceae*	8.02^a^	25.84^b^	17.07^ab^	4.10	0.0395
*Lachnospiraceae*	1.87^b^	1.00^ab^	0.71^a^	0.28	0.0391
*Lactobacillaceae*	12.76^a^	8.52^a^	24.62^b^	1.90	0.0006
*Leuconostocaceae*	3.09^a^	2.63^a^	7.55^b^	0.74	0.0020
*Microbacteriaceae*	0.25	1.46	1.05	0.47	0.2370
*Micrococcaceae*	4.10	3.09	2.75	0.47	0.1608
*Nocardioidaceae*	1.13	1.82	2.21	1.07	0.7790
*Peptostreptococcaceae*	1.61	1.29	0.87	0.22	0.1073
*Rickettsiaceae*	1.30^b^	0.68^a^	0.20^a^	0.12	0.0005
*Rivulariaceae*	8.10^b^	6.16^b^	1.77^a^	1.05	0.0060
*Ruminococcaceae*	1.11	0.64	0.42	0.21	0.1072
*Streptococcaceae*	10.70	7.23	11.07	0.99	0.0431
203 families^*^	9.27	9.52	6.80	0.96	0.1435

Values in the same row having different superscript letters are significantly different at *p* < 0.05, (*n* = 4)

^*^Grouped data of the rest of classes, orders and families of bacteria with values lower than 0.5% plus the unidentified groups

Five hundred forty-one genera were identified, which represented the 85.10 ± 3.51% of the total samples. After comparing the most predominant genera present in fish digesta (Fig. 3), sea trout fed the CON diet presented higher amounts of *Clostridium* and *Lactobacillus*, and the highest content was observed for *Enterococcus* followed by *Clostridium* in the MW group, but the most representative genera in the SW group were *Pediococcus* and *Enterococcus*.

The total amount of bacterial species identified varied among treatment groups. In the CON diet group, only 67.75 ± 4.63% of the bacteria were identified, whereas the MW diet group exhibited the highest percentage of identified species at 73.25 ± 3.36%, followed by the SW diet group at 70.07 ± 7.30%. When observing the most predominant species in each treatment group (Table 4), the fish fed the CON diet presented the highest percentage of *Clostridium cadaveris* and *Calothrix parietina.* In the case of the MW group, the most abundant species were also *C. cadaveris* and *C*. *parietina* along with *Enterococcus silesiacus*. Moreover, the highest abundances were observed for *Pediococcus pentosaceus*, *C. cadaveris*, and *Enterococcus durans*. In addition, significant differences were detected in the SW group, with the lowest levels observed for *Alkaliphilus crotonatoxidans, C. parietina, C. cadaveris, Lactobacillus antri, L. delbrueckii*, and *Streptococcus gallinaceus*, and the highest values observed for *E. durans, E. gallinarum*, *E. gilvus*, *Lactococcus garvieae, P. pentosaceus, P. acidilactici, P. stilesii* and *Weisella cibaria* (*p* < 0.05)*.*

**Table 4:** ANOVA results of the most representative bacterial phylotypes (%) isolated from the gut digesta of sea trout fed with the three experimental diets: control diet (CON), mealworm diet (MW), and superworm diet (SW)

Phyla	Species	CON	MW	SW	SEM	*P*-value
*Firmicutes*	*Alkaliphilus crotonatoxidans*	4.04^b^	3.38^b^	1.75^a^	0.47	0.0186
*Cyanobacteria*	*Calothrix parietina*	8.10^b^	6.15^b^	1.77^a^	1.05	0.0060
*Firmicutes*	*Clostridium cadaveris*	15.47^b^	12.96^b^	5.94^a^	1.69	0.0085
*Firmicutes*	*Enterococcus avium*	4.49	5.58	2.90	0.87	0.1455
*Firmicutes*	*Enterococcus casseliflavus*	0.66	0.69	0.51	0.10	0.4619
*Firmicutes*	*Enterococcus durans*	0.30^a^	1.16^a^	5.78^b^	0.63	0.0004
*Firmicutes*	*Enterococcus gallinarum*	0.05^a^	0.25^a^	1.08^b^	0.10	0.0001
*Firmicutes*	*Enterococcus gilvus*	0.51^a^	0.64^a^	2.20^b^	0.23	0.0010
*Firmicutes*	*Enterococcus lactis*	0.18^a^	2.69^b^	2.60^b^	0.50	0.0095
*Firmicutes*	*Enterococcus mundtii*	0.004	2.10	0.04	0.90	0.2225
*Firmicutes*	*Enterococcus silesiacus*	0.01	7.39	0.07	3.00	0.1909
*Proteobacteria*	*Escherichia albertii*	1.53	0.77	2.10	1.08	0.6909
*Firmicutes*	*Lactobacillus antri*	1.35^b^	1.14^b^	0.52^a^	0.14	0.0064
*Firmicutes*	*Lactobacillus delbrueckii*	2.41^b^	0.11^a^	0.30^a^	0.41	0.0054
*Firmicutes*	*Lactobacillus oris*	2.32^b^	1.94^ab^	0.80^a^	0.30	0.0156
*Firmicutes*	*Lactococcus garvieae*	1.36^a^	1.23^a^	3.93^b^	0.48	0.0049
*Firmicutes*	*Pediococcus acidilactici*	0.06^a^	0.12^a^	2.24^b^	0.21	0.0001
*Firmicutes*	*Pediococcus pentosaceus*	0.15^a^	0.32^a^	8.80^b^	0.84	0.0001
*Firmicutes*	*Pediococcus stilesii*	0.04^a^	0.08^a^	1.89^b^	0.16	0.0001
*Firmicutes*	*Streptococcus gallinaceus*	2.80^b^	1.83^ab^	1.56^a^	0.30	0.0380
*Firmicutes*	*Weissella cibaria*	1.52^a^	1.42^a^	5.39^b^	0.55	0.0009
*Firmicutes*, *Cyanobacteria*, *Proteobacteria*, and *Actinobacteria*	1307 spp	20.39	20.33	18.48	1.51	0.6123

Values in the same row having different superscript letters are significantly different at *p* < 0.05, (*n* = 4)

## Discussion

In the last several decades, the importance of the gut microbiota has been documented in numerous studies showing that growth performance and fish health are closely related to the microbiota. As Butt and Volkoff (2019) commented, feeding habits influence the structure and composition of the gut microbiota [2]. Additionally, plant-based proteins change the content and structure of the autochthonous microbiota of carnivorous species such as sea trout; in contrast, the use of a natural source of protein such as insect meals may play a role in maintaining the amount of these types of microorganisms that are part of the gut environment of the fish and enhance fish health [12]. Furthermore, insect meals would be able to modulate the microbiota of these animals due to the chitin and antimicrobial peptide contents [14, 15].

In this trial, more than 40% of FM (fish meal) was replaced with insect meals in the two diets, although the insect meals produced similar growth and survival rates when observing the weight gain. When analyzing the bacteria present in the digesta, the dominant phylum in all the treatment groups was Firmicutes, in contrast to brown trout fed a commercial diet, in which the dominant phylum was Proteobacteria, ranging from 88.4 to 92.6% [11]. However, Michl et al. (2019) reported a reduction in the amount of *Proteobacteria* and an increase in the amounts of *Firmicutes* and *Bacteriodetes* in the same species fed diets with more plant-based meals [7]. In contrast, Rimoldi et al. (2019) detected a gradual increase in the abundance of *Tenericutes* and a reduction in the abundances of *Proteobacteria* and *Firmicutes* in rainbow trout fed different amounts of black soldier fly meals [16]. Moreover, Kononova et al. (2019) affirmed that *Proteobacteria* is more abundant in carnivorous species and *Firmicutes* is more abundant in herbivorous species [12]. The results from the present trial showed that the abundance of *Firmicutes* would be conditioned by the amount of plant meal in the three diets, which was approximately 47% of the total, but not the inclusion of insect meals.

After performing a detailed analysis of the classes present in the digesta, *Bacilli* was the most abundant in all treatment groups, followed by Clostridia, both of which belong to the *Firmicutes* phylum, but the sum of *Alphaproteobacteria* and *Gammaproteobacteria*, which belong to the *Proteobacteria* phylum, presented similar amounts in all treatment groups, indicating that MW and SW meals exerted the same effect as FM on the digesta of sea trouts. In addition, the phylum *Firmicutes* and class *Clostridia* have been repeatedly identified in the digestive tracts of herbivorous fish, and as described above, the higher abundance of this class would be related to the higher amount of plant meal present in the diets [5].

The order and family distribution followed a similar trend as the class distribution. Although the bacterial genera exhibited changes based on the type of protein meal source, the most representative genera in the CON group were *Clostridium* and *Lactobacillus* and those in the MW group were *Enterococcus* and *Clostridium*, but the most representative genera in the SW group were *Pediococcus* and *Enterococcus*. The bacterial genera exhibited increased in that study are used as probiotics in aquaculture, increasing bacterial diversity [2], which probably occurred in fish fed these insect meals. The type of meals exerted a direct effect on the abundance of different genera in the intestinal digesta.

An analysis of the species abundance showed that fish fed the diet with SM exhibited a decrease in the relative abundance of *C. cadaveris* compared to the CON and MW groups; this species is known as a component of the normal fecal flora of humans and animals, which affects people with a poor overall condition of immunosuppression [17]. On the other hand, *C. cadaveris* is one of the most prominent bacterium present during the decay of dead bodies [17]. Moreover, *C. cadaveris* might trigger bacteremia that is related to a high mortality rate in humans. In the present study, the relative abundance of the commensal species *C. cadaveris* was decreased in the SW group, but significant differences were not observed between the CON and MW groups. Therefore, the reduction in the relative abundance *C. cadaveris* in the gastrointestinal tract of fish induced by the diet containing SW may be considered as a positive effect on public health.

*C. parietina* belongs to phylum *Cyanobacterium* and was previously detected in alkaline and oxygenated freshwaters [18]. The growth of *Cyanobacteria* is stimulated by the hypoxia of water reservoirs. Moreover, the contamination of dry food and feed with *Cyanobacterium* is considered a risk of toxin prevalence. Moreover, *C. parietina* is the bacteria with a higher potential for endotoxin production. The diet containing *Zoophobas morio* caused a decrease in the abundance of the bacterial species *C. parietina* in the fish GIT, which may reduce possible cyanobacterial toxin reservoirs in the fish GIT.

The diet containing SW improved the commensal probiotic microbiome in intestinal digesta of *Salmo trutta* vr. *trutta*. The SW diet increased the abundance of some bacterial genera, such as *Pediococcus,* which is considered a bacteria that can positively affect GIT of fish. *Pediococcus* is a genus of gram-positive lactic acid-producing bacteria belonging to the family Lactobacillaceae. *Pediococcus pentosaceus* genus is considered as a promising strain for both the food industry and biological applications [19]. The mechanism of action includes bacteriocins (pediocins) or bacteriocin-like substances (BLISs). It has been shown that *P. pentosaceus* can inhibit the growth of bacteria such as *Staphylococcus* *aureus, Enterococcus* *faecalis, Clostridium perfringens, Shigella flexneri, Salmonella enteritidis*, *Listeria monocytogenes,* and *Escherichia coli* [19–24]. The inhibiting effect was also observed against fungi especially *Aspergillus*, *Penicillium*, *Fusarium*, and *Candida species* [25–28].

In the SW group, an increase in the abundance of pediococci was observed, with the most abundant species identified as *P. pentosaceus* in the SW group. The bacterial species *P. pentosaceus* exerts bacteriocynogenic effects against some pathogenic bacteria [29]. Moreover *P. pentosaceus* regulate environmental homeostasis enforcing systemic immunity and enhancing anti-inflammatory ability [19].

*Enterococcus* is a key component of the intestinal flora of humans and is widespread in the intestines of most animals, including fish. Some species belonging to the *Enterococcus* genus, such as *Enterococcus faecalis* from fish intestine, are used as aquatic probiotics [30]. The SM diet increased the abundance of some Enterococcus species in the fecal digesta, among which *E. durans* may be considered a possible probiotic, because it potentially produces bacteriocins, namely, durancins [31]. Another species with probiotic potential that have been isolated from fish is *Enterococcus gallinarum* that regulates the innate immune response [32]. An increase in the abundance of *Enterococcus gilvus* was also observed in the fecal digesta of the analyzed SW group*.* The analysis of gene expression in *Enterococcus gilvus* has identified novel carotenoid biosynthesis genes that improve the multistress tolerance of *Lactococcus lactis* and promotes their activity toward methicillin-resistant *S. aureus* (MRSA) and vancomycin-resistant enterococci (VRE) [33]. Additionally, *W. cibaria*, which was more abundant in the SW group, has shown to be an effective probiotic in hybrid surubim [34]. Although significant differences among certain groups of bacteria were observed, the composition of the most representative species shows that they are part of the digestive tract flora, the environment, or part of the protein sources with probiotic properties that help the fish to thrive and achieve target growth and survival rates. In addition, Gajardo et al. (2016) commented that LAB are more abundant in salmon fed a plant-based diet than in fish fed a fishmeal-based diet [35], although, Ringø and Gatesoupe (1998) commented that LAB, such as the *Lactobacillus, Carnobacterium*, and *Streptococcus* genera, are also commonly detected in healthy fish microbiota of different fish species, including salmonids [36]. However, insect meals also increase the amount of LAB, as observed in the present study.

Furthermore, when comparing alpha diversity parameters, the inclusion of insect meal in the diet did not modify the different parameters measured, such as richness, evenness, and dominance. The Shannon H values were similar to those obtained in rainbow trout fed only FM and greater than 60% of *Tenebrio molitor* meal [13]. Additionally, the Chao1 values obtained in the present study were similar to those observed in the digesta of the proximal intestine of salmon fed 45% FM and 38% plant meals [35]. These authors obtained a higher Shannon H index than observed in our results. Moreover, in brown trout fries fed three experimental diets, 100% FM, 50% and 90% plant-based diets followed by a crossover feeding design, plant-based diets produced higher Chao1 and Shannon indexes than the FM diet, although the Chao1 values were lower than the values reported for sea trout in this experiment [7]. In general, the diversity among treatments was similar. Additionally, the NMDS analysis and the similarities of the clusters showed that the microbiome of the *Tenebrio molitor* group is more similar to the FM group than the *Zoophobas morio* group, which would be more useful for salmonid nutrition, as described by Antonopoulou et al. (2019) [13].

As mentioned above, the two insect meals exerted a similar effect to FM on maintaining the alpha diversity, and the values of dominance, equitability, and evenness were similar between all treatment groups, showing a balanced microbiome population that varied in abundance among bacterial classes, orders, genus and species as a natural consequence of the type of protein sources used. Nevertheless, the different meals that the fish consumed exerted positive effects on the microbiome, growth and survival performance of the sea trout, although the predominance of phylum *Firmicutes* in all treatment groups would be a consequence of the amount of plant meals, which were higher (47.17%) than animal meals (33.5%) in the diets, particularly for soybean meal, as highlighted by Kononova et al. (2019) and Michl et al. (2019) [7, 12]. Nevertheless, we cannot forget that plant meals are part of all commercial diets because of their availability and lower prices than fishmeal, and they are used to study the effects of alternative meals, such as insect meals.

## Conclusion

Insects are part of the sea trout diet in nature, at least in the first stages of development, before these fish feed on more diverse prey, including other fish. To conclude, this finding may explain why the main phyla present in the digesta were similar in all the treatment groups. However, the effect of each type of meal on the lower taxonomic levels was evident, particularly in the case of superworm meal. These differences were highlighted through the NMDS and the clusters, where fish fed mealworm meal were more related to fish fed fishmeal than fish fed superworm meal. Nevertheless, further studies are necessary to corroborate the finding that insect meals are one of the best alternatives to replace fishmeal in the diets of carnivorous fish.

## Methods

### Fish rearing conditions and experimental diets

Live insects were provided by HiProMine S.A (Robakowo, Poland). The larvae were euthanized by freezing at -20 °C for 24 h, after which the insects were oven-dried at 50 °C for 24 h and finely ground. Then, two commercial proteases were used to hydrolyze the dried larvae meals in two subsequent steps. The full-fat larvae meals were ground and mixed using distilled water at a ratio of 4:1 (w:v) to achieve a consistency suitable for enzyme hydrolysis. Initially, the diluted bacterial (*Bacillus amyloliquefaciens*) endopeptidase enzyme Corolase® 7090 (AB Enzymes GmbH, Darmstadt, Germany) was added to the meals at a concentration of 1.5 g·kg − 1 of protein, and the mixture was heated for five hours at 50 °C, according to the manufacturer’s instructions. Next, 0.75 g·kg − 1 of the fungal protease enzyme Flavourzyme® (endopeptidase and exopeptidase from *Aspergillus oryzae*; supplied by Novozymes A/S, Denmark) was added to the mixture, which was homogenized and hydrolyzed for three hours. The hydrolyzed meals were kept at 4 °C until diet preparation.

A control diet (CON) and two experimental diets were formulated. A fixed 10% of hydrolyzed insect meal was included in both experimental diets, corresponding to 42% and 44% of fishmeal replacement by hydrolyzed mealworm (MW) and superworm (SW) diets, respectively.

The diets were manufactured at the Aquaculture Experimental Station in Muchocin, Poland using a semi-industrial single-screw extruder (Metalchem S-60, Gliwice, Poland) at 110 °C. Each feed was produced in two pellet sizes with 1.5-mm diameter used from 1st to 30th day of the experiment and 2.5-mm used from 31st to 60th day.

After extrusion, pellets were dried in an oven for 48 h at 40 °C, and then fish oil was added to the mildly heated pellets. The nutritional values are shown in Table 5.

**Table 5:** Chemical composition of the three experimental diets: fishmeal diet, enzyme hydrolysed mealworm diet and enzyme hydrolysed superworm diet

Ingredients (g kg^−1^)	Diets		
	**CON**^**a**^	**MW**^**b**^** (42%)**	**SW**^**c**^** (44%)**
Fish meal	250	145	140
Mealworm meal^a^	-	100	-
Superworm meal^b^	-	-	100
Soybean meal	100	100	100
Wheat flour	219	220	226
Corn gluten	150	150	150
Blood meal	70	100	100
Brewer yeast	35	35	35
Fish oil	164	143	140
Dicalcium phosphate	7.2	0.8	2.1
Premix^c^	1.5	1.5	1.5
DL-Methionine	1.2	2.2	2.4
L-Lysine HCL	1.1	1.8	2.0
L-Threonine	0.6	0.6	0.7
**Proximate analysis (% DM)**			
Dry matter	93.0	93.7	93.5
Crude protein	48.0	51.1	49.8
Crude lipid	16.3	14.6	15.3
Ash	6.5	5.4	5.1
Crude fibre	1.7	1.7	1.7
Chitin^d^	0	9.3	4.8
NFE^e^	35.0	33.8	35.0
Gross energy (MJ kg^−1^)	22.18	22.77	22.55

Fishmeal diet (CON)

Enzyme hydrolysed mealworm diet (MW)

Enzyme hydrolysed superworm diet (SW)

^a^Mealworm meal (dry matter: 95.58%, crude protein: 47.0%, crude lipid: 29.6%)

^b^Superworm meal (dry matter: 96.32%, crude protein: 49.3%, crude lipid: 33.6%)

^c^Polfamix BASF Poland Ltd. (Kutno, Poland) (g kg^−1^): vitamin A, 1 000 000 IU; vitamin D_3_, 200 000 IU; vitamin E, 1.5 g; vitamin K, 0.2 g; vitamin B_1_, 0.05 g; vitamin B_2_, 0.4 g; vitamin B_12_, 0.001 g; nicotinic acid, 2.5 g; D-calcium pantothenate, 1.0 g; choline chloride, 7.5 g; folic acid, 0.1 g; methionine, 150.0 g; lysine, 150.0 g; Fe, 2.5 g; Mn, 6.5 g; Cu, 0.8 g; Co, 0.04 g; Zn, 4.0 g; J, 0.008 g; carrier > 1000.0 g

^d^Calculated based on chitin content of insect meals

^e^Nitrogen-free extract = 1,000 – (crude protein + ether extract + crude fibre + ash)

Sea trout were transported from the Feed Production Technology and Aquaculture Experimental Station in Muchocin, Poland, to the Division of Inland Fisheries and Aquaculture laboratory where the experiment was conducted.

The diets were manufactured at the Aquaculture Experimental Station in Muchocin, Poland using a semi-industrial single-screw extruder (Metalchem S-60, Gliwice, Poland) at 110 °C. Each feed was produced in two pellet sizes with 1.5-mm diameter used from 1st to 30th day of the experiment and 2.5-mm used from 31st to 60th day.

At the beginning of the experimental period, a total of 225 sea trout fingerlings (5.08 ± 0.9 g) were distributed into nine tanks. The fiberglass tanks with a 60-L capacity were supplied with water from the reservoir in an open-flow system at a rate of 2 L min^−1^. Water parameters were recorded daily. The temperature was 14.7 ± 0.6 °C, the dissolved oxygen content was maintained at a constant value of 7.5 ± 0.3 mg L^−1^ and the photoperiod was maintained at 16:8 (light:dark) during the entire experiment. The fish were weighed individually at the beginning and the end of study to measure the body weight gain (BWG) using the following formula: BWG (g) = final body weight (g) − initial body weight (g). The survival rate (SR) was calculated using the formula: SR (%) = (final number of live fish/initial number of live fish) × 100.

During the experiment, the animals were weighed and counted every two weeks to adjust the feed intake ratio, and the growth and feed efficiency parameters were recorded. The feed ratio was based on a feeding chart designed for Atlantic salmon, taking into consideration the average body weight of the fingerlings and the water temperature. Animals were fed with automatic band feeders for 12 h per day, 7 days a week. The mean feed ratios were in the range from 1.39% to 1,48% of fish biomass daily depending on feed consumption.

The fish were housed in the experimental tanks for 60 days, after which the animals were sacrificed by an overdose of clove oil, according to the EU (no 2010/63/EU) regulation for experimental animals. Under sterile conditions, the animals were dissected and the digesta from the distal part of the intestine were collected. The samples were pooled by 4 fish and immediately packed, sealed in sterilized plastic bags, and stored at − 80 °C for analyses of the microbial populations by next-generation sequencing (NGS).

### DNA extraction

The research was conducted in accordance with the methodology of the Authors' previous research [37, 38]. DNA was extracted with a commercial kit (Sherlock AX, A&A Biotechnology, Poland) according to the manufacturer’s instructions.

### Library preparation, sequencing, basic analysis

An analysis of the bacterial population was performed based on the hypervariable region V3-V4 of the 16S rRNA gene. The specific sequences of the 341F and 785R primers with adaptors (341F primer 5′ TCGTCGGCAGCGTCAGATGTGTATAAGAGACAGCCTACGGGNGGCWGCAG; 785R primer 5′ GTCTCGTGGGCTCGGAGATGTGTATAAGAGACAGGACTACHVGGGTATCTAATCC) were used to amplify the selected region and prepare the library. The primers contain Illumina adaptor sequence (in italics) and V3-V4 16S rRNA locus specific sequence [39]. All steps including amplification, indexing and library quantification were performed according to the protocol "16S Metagenomic Sequencing Library Preparation" (Illumina, USA). Briefly, the PCR reaction was performed with Q5 Hot Start High-Fidelity 2X Master Mix (New England Biolabs, UK) under the following reaction conditions: 95 °C for 3 min; 25 cycles of: 95 °C for 30 s, 55 °C for 30 s, 72 °C for 30 s, 72 °C for 5 min, Hold at 4°. The resulting amplicons were then indexed with Nextera XT Index Kit (Illumina, USA). Library size was evaluated on Bioanalyzer 2100 DNA High Sensitivity chip (Agilent). The library was validated to the expected size on a Bioanalyzer trace for the final library of ~ 630 bp. The libraries were quantified using a fluorometric quantification method using dsDNA binding dyes. Individual concentrations of DNA libraries were calculated in nM, based on the size of DNA amplicons, as determined by an Agilent Technologies 2100 Bioanalyzer [40, 41].

Sequencing was performed on the MiSeq, 2 × 250 PE (paired-end) in order to obtain at least 50 000 read pairs per sample. Automatic data analysis was performed on the MiSeq apparatus, using the MiSeq Reporter (MSR) v2.6 software and 16S Metagenomics app. The analysis consisted of three stages: automatic demultiplexing of samples, generating fastq files containing raw reads and reads classification at several taxonomic levels: kingdom, phylum, class, order, family, genus, and species. 16S Metagenomics app provides taxonomic classification to species level based on the SILVA reference sequence database [42, 43].

### Statistical analysis

Alpha diversity was analyzed in the QIIME. The beta diversity measure was calculated based on the Bray–Curtis method [44]. Bacterial diversity was assessed by Shepard analyses. Nonmetric multidimensional scaling (NMDS) has been performed to analyze bacterial community composition.

The Kolmogorov–Smirnov test was used to determine the normality of the data distribution and equality of variances. Data are presented as the means ± standard errors of the means (SEM). Statistical significance was declared at *p* ≤ 0.05. Bioinformatic analysis ensuring the classification of readings by species level was carried out with the free Infostat software was used for the one-way ANOVA, and if significant differences were observed among treatment groups, data were further analyzed using Tukey’s post hoc test.

## Data Availability

All data are included in this published article. The raw datasets are available from the corresponding author on reasonable request.

## Abbreviations

NGS: Next generation sequencing
CON: Control diet
MW: Mealworm diet
SW: Superworm diet
FM: Fish meal
DM: Dry matter
NMDS: Non-metric Multi-dimensional Scaling
